# Production of hydroxycinnamoyl anthranilates from glucose in *Escherichia coli*

**DOI:** 10.1186/1475-2859-12-62

**Published:** 2013-06-28

**Authors:** Aymerick Eudes, Darmawi Juminaga, Edward E K Baidoo, F William Collins, Jay D Keasling, Dominique Loqué

**Affiliations:** 1 Joint BioEnergy Institute, Emeryville, CA 94608, USA; 2 Physical Biosciences Division, Lawrence Berkeley National Laboratory, Berkeley, CA 94720, USA; 3 California Institute for Quantitative Biosciences and the Synthetic Biology Institute at UC Berkeley, Berkeley, CA 94720, USA; 4 Eastern Cereal and Oilseed Research Centre, Agriculture and Agri-Food, Ottawa, ON K1A 0C5, Canada; 5 Department of Bioengineering, Department of Chemical & Biomolecular Engineering, University of California, Berkeley, CA 94720, USA

**Keywords:** Avenanthramide, Tranilast, BAHD, Antioxidant, Anti-inflammatory, Tyrosine, Anthranilate, Hydroxycinnamate, Biological synthesis, *Escherichia coli*

## Abstract

**Background:**

Oats contain hydroxycinnamoyl anthranilates, also named avenanthramides (Avn), which have beneficial health properties because of their antioxidant, anti-inflammatory, and antiproliferative effects. The microbial production of hydroxycinnamoyl anthranilates is an eco-friendly alternative to chemical synthesis or purification from plant sources. We recently demonstrated in yeast (*Saccharomyces cerevisiae*) that coexpression of 4-coumarate: CoA ligase (4CL) from *Arabidopsis thaliana* and hydroxycinnamoyl/benzoyl-CoA/anthranilate N-hydroxycinnamoyl/benzoyltransferase (HCBT) from *Dianthus caryophyllus*enabled the biological production of several cinnamoyl anthranilates upon feeding with anthranilate and various cinnamates. Using engineering strategies to overproduce anthranilate and hydroxycinnamates, we describe here an entire pathway for the microbial synthesis of two Avns from glucose in *Escherichia coli*.

**Results:**

We first showed that coexpression of HCBT and Nt4CL1 from tobacco in the *E*. *coli* anthranilate-accumulating strain W3110 *trpD9923* allowed the production of Avn D [*N*-(4′-hydroxycinnamoyl)-anthranilic acid] and Avn F [*N*-(3′,4′-dihydroxycinnamoyl)-anthranilic acid] upon feeding with *p*-coumarate and caffeate, respectively. Moreover, additional expression in this strain of a tyrosine ammonia-lyase from *Rhodotorula glutinis* (*Rg*TAL) led to the conversion of endogenous tyrosine into *p*-coumarate and resulted in the production of Avn D from glucose. Second, a 135-fold improvement in Avn D titer was achieved by boosting tyrosine production using two plasmids that express the eleven genes necessary for tyrosine synthesis from erythrose 4-phosphate and phosphoenolpyruvate. Finally, expression of either the *p*-coumarate 3-hydroxylase Sam5 from *Saccharothrix espanensis* or the hydroxylase complex HpaBC from *E*. *coli* resulted in the endogenous production of caffeate and biosynthesis of Avn F.

**Conclusion:**

We established a biosynthetic pathway for the microbial production of valuable hydroxycinnamoyl anthranilates from an inexpensive carbon source. The proposed pathway will serve as a platform for further engineering toward economical and sustainable bioproduction of these pharmaceuticals and other related aromatic compounds.

## Background

**Figure 1:**
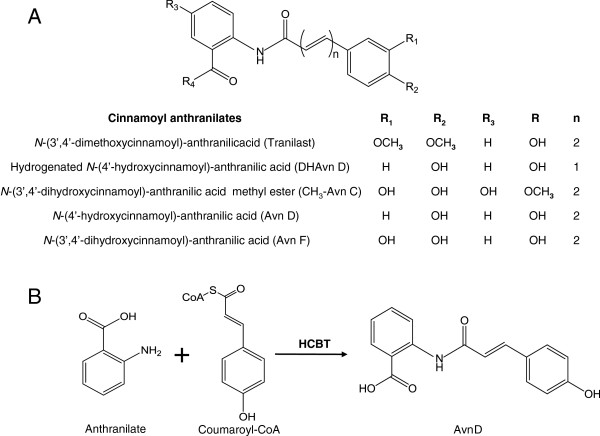
**General structure of cinnamoyl anthranilates. (A)** Tranilast, DHAvn D, and CH_3_-Avn C are represented. The two cinnamoyl anthranilates (Avn D and Avn F) produced biologically in this study are shown n=1, single carbon bond; n=2, double carbon bond. **(B)** Enzymatic reaction catalyzed by HCBT for the synthesis of Avn D.

Hydroxycinnamoyl anthranilates are part of the large cinnamoyl anthranilates family, a class of molecules with beneficial health properties. For example, avenanthramides (Avns) are natural hydroxycinnamoyl anthranilates found in oats (*Avena sativa* L.) at low concentrations (a few parts per million in grains) and whose antioxidant, anti-inflammatory, and antiproliferative effects are considered to contribute to the health benefits of oatmeal consumption [1-6]. The antioxidant effects of Avns have been established in several animal studies [7-9], and their anti-inflammatory properties illustrated in model systems of atherosclerosis, diabetes, itching, and breast cancer. In particular, Avn-enriched oat extracts, synthetic dihydroavenanthramide D (DHAvn D) and Avn C methyl ester (CH_3_-Avn C) (Figure 1A) were shown to inhibit the activation of the NF-κB transcription factor, which is a master regulator of infection and inflammation [10-14]. Consequently, DHAvn D has been developed as a drug to reduce histamine-related skin disorders [15]. Lastly, the antiproliferative effects of Avn have been demonstrated on vascular muscle and colonic cancer cell lines [16-18]. Similarly, tranilast [*N*-(3′4′-dimethoxycinnamoyl)-anthranilic acid] (Figure 1A) is a synthetic cinnamoyl anthranilate used as an antihistamine in Japan and South Korea for the treatment of allergic disorders, hypertrophic scars, and keloids [19-21]. Tranilast has anti-inflammatory and antiproliferative effects and is currently evaluated clinically for the treatment of multiple sclerosis and various arthritic conditions [22-24]. Moreover, the antitumor potential of tranilast has been evidenced in several clinical trials [25], and the design of analogues that exhibit higher anti-fibrotic activity has been extensively investigated [26-30].

Using microbes for biological synthesis of therapeutic drugs or precursors offers an alternative production strategy to commonly employed methods such as direct extraction from source organisms or chemical synthesis. Microbial expression systems have several advantages such as reduced requirements for toxic chemicals and natural resources; consistent quality; scalability; simple extraction; and potential for higher synthesis efficiency [31,32]. Taking into consideration the expanding number of therapeutic applications for cinnamoyl anthranilates, as well as the fact that these molecules are currently synthesized chemically or extracted from food sources [33,34], we attempted to design a pathway for their de novo production from glucose using *E*. *coli* as a production platform.

HCBT is an acetyltransferase from the BAHD family [35], which couples *p*-coumaroyl-CoA with anthranilate via an amide bond to produce Avn D (Figure 1B) [36]; while 4CL enzymes convert cinnamates into their corresponding CoA thioesters [37]. We previously engineered a yeast strain that coexpresses 4CL and HCBT for the production of several cinnamoyl anthranilates, such as Avn D and Avn F, upon feedings with anthranilate and various cinnamates. This highlighted the potential of using these enzymes for the biological production of cinnamoyl anthranilates [38]. *E*. *coli* is a host of choice for the expression of complex pathways and the production of elaborate molecules such as aromatic compounds from cheap carbon sources [39,40].

**Figure 2:**
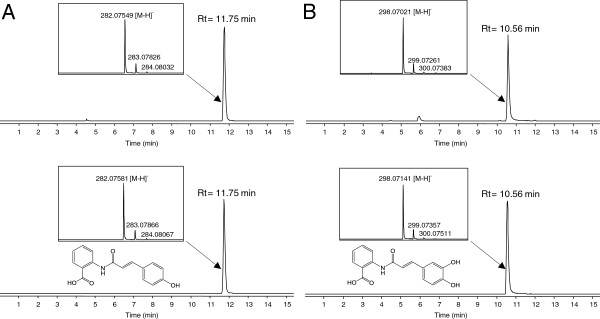
**Production of Avn D and Avn F in engineered *E*. *coli *coexpressing HCBT and Nt4CL1.** Detection of Avn D **(A)** and Avn F **(B)** from the culture medium of the W3110 *trpD9923* strain harboring the pAvn vector and fed with *p*-coumarate and caffeate (300 μM), respectively. ESI-MS spectra were obtained after LC-TOF MS analysis of the culture medium of engineered *E*. *coli* strains (upper panels) and of authentic standard solutions (lower panels).

In this study, we primarily focused on the biological synthesis of Avn D, which features a basal core structure of hydroxycinnamoyl anthranilates. For this purpose, a previously characterized *E*. *coli* anthranilate-accumulating strain was selected as a chassis [41,42]. In that strain, coexpression of Nt4CL1 and HCBT led to the production of Avn D and Avn F when the culture medium was supplemented only with *p*-coumarate and caffeate, respectively. This validated the functional expression and activity of both plant enzymes in our chassis. The production system was then affranchised from precursor feeding by additional expression of *Rg*TAL, which converts tyrosine into *p*-coumarate [43,44] (Figure 2). Avn D biosynthesis was further enhanced by expressing a two-plasmid-based modular biosynthetic pathway for tyrosine overproduction from glucose [45]. Finally, Avn F was also biologically produced de novo upon expression of either Sam5 or HpaBC, which are two hydroxylases that convert *p*-coumarate into caffeate [46,47].

## Results and discussion

### Expression of Nt4CL1 and HCBT in *E*. *coli* strain W3110 *trpD9923*

*E*. *coli* W3110 *trpD9923* strain is a tryptophan auxotroph that over-accumulates anthranilate due to a nonsense mutation in the *trpD* gene, which abolishes anthranilate phosphoribosyltransferase activity but does not affect anthranilate synthase activity [41,42]. This strain was shown to be suitable for metabolic engineering because expression of genes from the shikimate pathway further increased anthranilate production [41]. We first constructed pAvn plasmid for coexpression of *Nt4CL1*, which encodes a 4CL that converts *p*-coumarate and caffeate into their corresponding CoA thioesters [48], and *HCBT*. To confirm that HCBT can catalyze the condensation of coumaroyl-CoA with anthranilate and produce Avn D in W3110 *trpD9923*, the strain was transformed with pAvn and grown in the presence of *p*-coumarate as a precursor. Cultures of W3110 *trpD9923* harboring an empty vector were also grown as a negative control. Only in the case of the strain expressing pAvn, LC-TOF MS analysis of the culture medium revealed a peak (Rt = 11.75 min) that corresponds to Avn D by comparison with an authentic standard solution (Figure 2A). Similarly, the engineered strain produced some Avn F (Rt = 10.56 min) when *p*-coumarate was substituted by caffeate in the medium (Figure 2B). This result confirms the affinity of HCBT for caffeoyl-CoA. It also demonstrates secretion of Avn outside of the production host, because the Avn D and Avn F content inside *E*. *coli* cells represented less than 5% of the amount quantified from the medium (data not shown).

### Biosynthesis of Avn D from glucose and titer improvement using a tyrosine overproduction strategy

**Figure 3:**
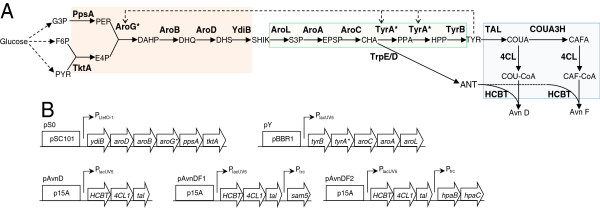
**Biosynthetic pathways for the microbial production of hydroxycinnamoyl anthranilates from glucose. (A)** Three compatible plasmids were used for the biological production of Avn D and Avn F from glucose. A first plasmid is a shikimate module which contains all the six genes for the production of shikimate (SHIK) from pyruvate (PYR), fructose 6-phosphate (F6P), and glyceraldehydes 3-phosphate (G3P) (orange box). A second plasmid is a tyrosine module which contains all the five genes for the production of tyrosine (TYR) from SHIK (green box). The third plasmid is the cinnamoyl anthranilate module which contains up to five genes for the conversion of TYR into two hydroxycinnamoyl-CoAs and their coupling to anthranilate (ANT) for the production of Avn D and Avn F (blue box).CAFA, caffeate; CAF-CoA, caffeoyl-CoA; CHA, chorismate;COUA, *p*-coumarate; COU-CoA, *p*-coumaroyl-CoA; DAHP, 3-deoxy-D-arabino-heptulosonate; DHQ, dehydroquinate; DHS, dehydroshikimate; E4P, erythrose 4-phosphate; EPSP, 5-enolpyruvoylshikimate 3-phosphate; HPP, 4 hydroxyphenlypyruvate; PEP, phosphoenolpyruvate; PPA, prephenate; and S3P, shikimate 3-phosphate. The enzymes (in bold face) are as follows: PpsA, phosphoenolpyruvate synthase; TktA, transketolase A; AroG, DAHP synthase; AroB, DHQ synthase; AroD, DHQ dehydratase; YdiB, shikimate dehydrogenase; AroL, shikimate kinase II; AroA, EPSP synthase; AroC, chorismate synthase; TrpE/D, anthranilate synthase I/II; TyrA, chorismatemutase/prephenate dehydrogenase; and TyrB, tyrosine aminotransferase; TAL, tyrosine ammonia-lyase; 4CL, *p*-coumarate:CoA ligase; COUA3H, *p*-coumarate 3-hydroxylase; HCBT, hydroxycinnamoyl/benzoyl-CoA/anthranilate N-hydroxycinnamoyl/benzoyltransferase. The dashed lines indicate where feedback inhibitions occur. Allosteric regulation of AroG and TyrA were removed by employing their respective feedback-resistant mutants, AroG* (D146N) and TyrA* (M53I; A354V), respectively. **(B)** Structures of the three plasmids used for cinnamoyl anthranilates production. The open blocks indicate the origins of replication, the open arrows represent the genes, and the angled arrows indicate the promoters.

To produce Avn D — without supplying costly precursors such as *p*-coumarate to the engineered *E*. *coli* strain — we designed a plasmid (pAvnD) that contains in a single operon *HCBT*, *Nt4CL1*, and a gene encoding *Rg*TAL for the conversion of tyrosine into *p*-coumarate (Figure 3A, B). Analysis of the culture medium of cells harboring pAvnD and grown for 24 hours revealed the presence of *p*-coumarate (1.0 ± 0.1 μM), which was produced from endogenous tyrosine upon *Rg*TAL activity, and a detectable amount of Avn D (~200 nM) (Table 1). Moreover, a 15% reduction of the final biomass density was observed for this strain compared to the control. We recently reported on a strategy for the overproduction in *E*. *coli* of tyrosine using two plasmids (pS0 and pY) that contain all the genes required for the synthesis of tyrosine from erythrose 4-phosphate and phosphoenolpyruvate (Figure 3A, B) [45]. As expected, this strategy applied to the W3110 *trpD9923* strain not only increased tyrosine titers (665-fold), but also enhanced anthranilate production (3.5-fold), since both metabolites are derived from chorismate via the shikimate pathway (Figure 3A; Table 1). This engineered strain, despite having a slower growth rate, showed no difference in final biomass density compared to that harboring empty vector controls. Furthermore, co-transformation of compatible shikimate (pS0) and tyrosine (pY) plasmids with pAvnD led to a 135-fold increase in extracellular Avn D (27.3 ± 0.1 μM) compared to the production achieved using pAvnD alone, after 24 h of culture (Table 1). The analysis of the culture medium also revealed that *p*-coumarate content (~32 μM) was much lower compared to that of tyrosine (~6 mM), suggesting that *Rg*TAL is a rate-limiting enzyme in the pathway (Table 1). As observed for the strain containing pAvnD alone, the strain harboring the three plasmids had a 15% reduction of the final biomass density.

**Table 1:** **Production of Avn D and precursors by engineered W3110 *trpD9923 E*. *coli *strains**

**Plasmids**	**Compounds (μM)**			
	**Anthranilate**	**Tyrosine**	***p*-Coumarate**	**Avn D**
Empty pZS21 + pBbB5a	1646 ± 63	9.3 ± 1.3	nd	nd
pS0 + pY	5878 ± 311	6201 ± 598	nd	nd
pAvnD	1564 ± 58	6.8 ± 0.9	1.0 ± 0.1	0.2 ± 0.1
pS0 + pY + pAvnD	5780 ± 251	5963 ± 219	32.6 ± 3.4	27.3 ± 1.4

Values are the means ± SD of five independent clones. *nd* not detected.

### Conversion of *p*-coumarate into caffeate and production of Avn F using Sam5

For the biological production of caffeate, and ultimately Avn F, we generated pAvnDF1 plasmid, which adds into the pAvnD backbone *sam5* under the control of the *trc* promoter (Figure 3A, B). Sam5 is a *p*-coumarate 3-hydroxylase that has been successfully expressed in *E*. *coli* for the biological synthesis of caffeate [49-51]. Expression of the genes harbored on pAvnDF1 plasmid in the W3110 *trpD9923* strain resulted in the production of a small amount of caffeate in the culture medium, but no Avn could be detected (Table 2). However, co-transformation of pAvnDF1 with pS0 and pY not only enhanced caffeate production (~230-fold), but also led to the biosynthesis of Avn F (~110 nM) in addition to Avn D (Table 2). No extracellular *p*-coumarate could be detected in these cultures, suggesting that most of it was efficiently converted into caffeate by Sam5.

**Table 2:** **Production of Avn F and precursors by engineered W3110 *trpD9923 E*. *coli *strains**

**Plasmids**	**Compounds (μM)**					
	**Anthranilate**	**Tyrosine**	***p*-Coumarate**	**Caffeate**	**Avn D**	**Avn F**
pAvnDF1	1498 ± 76	6.0 ± 1.0	nd	0.28 ± 0.07	nd	nd
pS0 + pY + pAvnDF1	5802 ± 298	6286 ± 150	nd	65.1 ± 8.3	0.07 ± 0.00	0.11 ± 0.04
pAvnDF2	1521 ± 44	2.0 ± 0.2	0.10 ± 0.01	0.13 ± 0.03	0.03 ± 0.00	nd
pS0 + pY + pAvnDF2	5644 ± 288	2503 ± 313	11.9 ± 2.2	14.9 ± 1.6	4.1 ± 0.7	0.54 ± 0.16

Values are the means ± SD of five independent clones. *nd* not detected.

**Figure 4:**
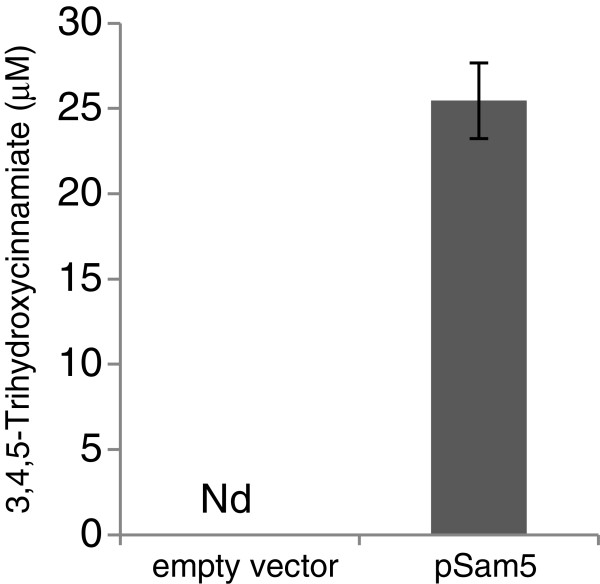
***In***-***vivo *enzyme activity of Sam5 towards caffeate.** Production of 3,4,5-trihydroxycinnamate detected in the culture medium of an *E*. *coli* strain expressing Sam5 and fed with caffeate. Error bars indicate mean values ± SD from three independent clones. Nd, not detected.

Interestingly, LC-TOF MS analysis revealed an additional new peak in the culture medium of the strains harboring pAvnDF1 and expressing Sam5. This peak was found to correspond to 3,4,5-trihydroxycinnamate based on the mass and elution time of an authentic standard (Additional file 1: Figure S1), and the 3,4,5-trihydroxycinnamate content represented ~ 1.6 μM in the culture medium of the pAvnDF1 containing strain and reached 48 μM when the tyrosine production pathway (pS0 + pY) was co-expressed in the pAvnDF1-containing strain. These observations strongly suggest that Sam5 can not only convert *p*-coumarate into caffeate, but also caffeate into 3,4,5-trihydroxycinnamate. To validate this hypothesis, an *E*. *coli* strain expressing Sam5 alone was grown in the presence of caffeate and the culture medium analyzed for the presence of 3,4,5-trihydroxycinnamate. Conclusively, this new compound was detected in the medium of the Sam5 strain but not in that of an empty vector control strain (Figure 4).This is, to our knowledge, the first report of an enzyme capable of hydroxylating caffeate. Although the conversion of caffeate into 3,4,5-trihydroxycinnamate is not desirable for the production of Avn F (and perhaps inhibitory for Nt4CL1 activity), this novel hydroxylating property for Sam5 presents an opportunity for the enzymatic synthesis of trihydroxylated cinnamoyl anthranilates. In this regard, we previously demonstrated in yeast the wide-range of substrate specificity for Arabidopsis 4CL5 and HCBT toward various substituted cinnamates and cinnamoyl-CoAs, respectively [38].

### Conversion of *p*-coumarate into caffeate and production of Avn F using the HpaBC complex

**Figure 5:**
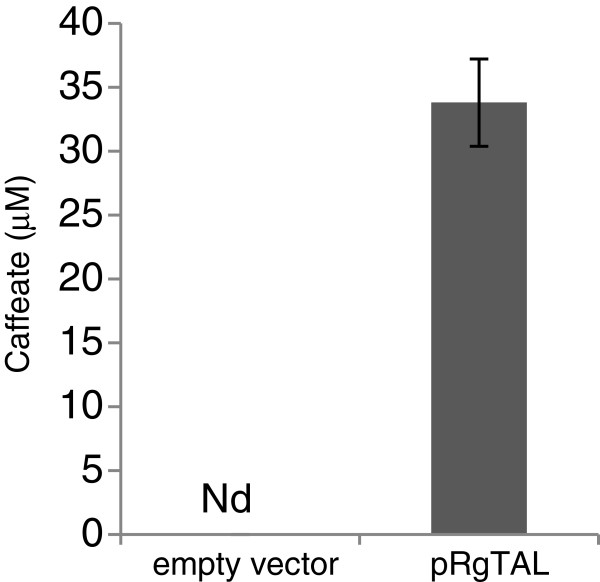
***In***-***vivo *enzyme activity of RgTAL towards L**-**dopa.** Production of caffeate detected in the culture medium of an *E*. *coli* strain expressing *Rg*TAL and fed with L-dopa. Error bars indicate mean values ± SD from three independent clones. Nd, not detected.

The enzyme complex consisting of a 4-hydroxyphenylacetate 3-hydroxylase (HpaB) and a flavin:NADH reductase (HpaC) from *E*. *coli* was tested for the biological production of caffeate and Avn F. The operon *hpaBC* is involved in 4-hydroxyphenylacetate degradation and several studies showed that the HpaBC enzyme complex can accept a broad range of substrates including tyrosine and *p*-coumarate [46,52,53]. We constructed a pAvnDF2 plasmid by placing the *hpaBC* operon under the control of the *trc* promoter into pAvnD plasmid (Figure 3B). Transformation of pAvnDF2 into *E*. *coli* W3110 *trpD9923* resulted in the production of small amount of caffeate in the culture medium, but only Avn D could be detected (Table 2). By contrast, co-transformation of pAvnDF2 with pS0 and pY enhanced caffeate production (~115-fold) and led to the biosynthesis of Avn F (~540 nM) in addition to Avn D (Table 2). Unlike the results of the biosynthesis of Avn F using Sam5, the expression of HpaBC maintained higher Avn D titers and did not produce any 3,4,5-trihydroxycinnamate nor entirely deplete *p*-coumarate content. This suggests that HpaBC is less efficient than Sam5 at converting *p*-coumarate into caffeate in our system, yet nevertheless Avn F titers using HpaBC were ~5-fold higher compared to those achieved using Sam5 (Table 2). Alternatively, the higher caffeate content and lower AvnF titers obtained using Sam5 could reflect a negative effect of 3,4,5-trihydroxycinnamate on 4CL1 activity. Moreover, we observed a reduction in tyrosine titers compared to those measured from the culturesof *E*. *coli* W3110 *trpD9923* harboring pAvnD or pAvnDF1. This was probably due to HpaBC activity, which can also convert tyrosine into L-dopa. Conclusively, we found that L-dopa concentration was ~4.4 mM in the culture medium of the pS0/pY/pAvnDF2 strain. Furthermore, based on previous studies showing that some tyrosine ammonia-lyases convert L-dopa into caffeate [46,54], an *E*. *coli* strain that expresses *Rg*TAL alone was created and grown in the presence of L-dopa. Interestingly, analysis of the culture medium of the *Rg*TAL strain revealed the presence of caffeate, which was absent in the medium of an empty vector control strain (Figure 5). These results demonstrate that *Rg*TAL exhibits some L-dopa ammonia-lyase activity and suggest that part of the caffeate produced in the strains harboring pAvnDF2 could be derived from L-dopa.

## Conclusions

This work is an example of biological production of valuable aromatic metabolites using a tyrosine-overproducing strategy applied to an anthranilate-accumulating strain. Considering the anthranilate titers achieved with the strain containing only the shikimate and tyrosine modules, the maximum theoretical yield for Avn D in this background would be ~5.8 mM. However, much lower Avn D titers were obtained for the strain harboring pS0, pY and pAvnD, probably due to poor conversion of tyrosine into *p*-coumarate as previously observed in various studies using heterologous expression of TALs [46,49,50], and potentially to the limited intracellular pools of coenzyme A availability [55]. It is particularly noteworthy that, because of its specificity to anthranilate as an acceptor, the BAHD acyltransferase HCBT allowed the exclusive biological synthesis of cinnamoyl anthranilates. For instance, no mass peaks corresponding to other phenylpropenoyl-amino acid amides consisting of a tryptophan, tyrosine or an L-dopa moiety — nor to hydroxycinnamate esters of shikimate or quinate — could be detected in the culture medium of our different *E*. *coli* Avn-producing strains.

The discovery that *Rg*TAL has L-dopa ammonia-lyase (DAL) activity is of interest and provides some opportunities for the design of new enzymes with a higher DAL/TAL activity ratio. In combination with tyrosine hydroxylase complexes such as HpaBC, such engineered DALs could be used to improve the bioproduction of caffeate from tyrosine via L-dopa and without generating *p*-coumarate as an intermediate, a competitive precursor for the biosynthesis of Avn F. Furthermore, the impact of expressing in our system 4CLs other than Nt4CL1 should be considered; especially in regard to production of Avn F, because Nt4CL1 is known to be less active with caffeate as a substrate compared to *p*-coumarate [56,57]. Finally, our rationally designed pathway can serve as a framework for improvement of Avn production using combinatorial approaches that have been shown previously to increase tyrosine production [58]. As an adjunct to the recent development of procedures that use safe methylating agents [59], this study describes a basis for eco-friendly production of cinnamoyl anthranilates such as Avn D and Avn F and can serve as a scaffold for the synthesis of more elaborate molecules such as tranilast and its analogs.

## Methods

### Chemicals and enzymes

The following chemicals and enzymes were used in this study: *p*-coumarate, L-tyrosine, anthranilate, L-dopa, isopropyl-β-D-thiogalactopyranoside (IPTG) (Sigma-Aldrich, St. Louis, MO, USA), caffeate (MPBiomedicals, Solon, OH, USA), 3,4,5-trihydroxycinnamate (Apin Chemicals Ltd, Abingdon, UK), restriction enzymes (NEB, Ipswich, MA, USA), PhusionHigh-Fidelity DNA Polymerase (Thermo Scientific, Waltham, MA, USA), Rapid DNA ligase Kit (Roche Applied Science, Indianapolis, IN, USA). All the enzymes were used in accordance with instructions provided by the manufacturers. *N*-(4′-hydroxy-(*E*)-cinnamoyl)-anthranilate (Avn D) and *N*-(3′,4′-dihydroxy-(*E*)-cinnamoyl)-anthranilate (Avn F) were prepared as described [1,38].

### Strains, plasmids, media, and growth conditions

*E*. *coli* DH10B (Life technologies, Foster City, CA, USA) was used for gene cloning and plasmid propagation. Bacterial strains and plasmids used in this study are described in Table 3. *E*. *coli* strain W3110 *trpD9923* was obtained from the *E*. *coli* Genetic Stock Center (Yale University, New Haven, CT). *E*. *coli* cells for gene cloning and plasmid propagation were grown in Luria-Bertani (LB) medium at 37°C. For cinnamoyl anthranilate production, *E*. *coli* W3110 *trpD9923* was used and cultured at 37°C in MOPS (morpholinepropanesulfonic acid)-M9 minimal medium [60] containing 1% glucose, 10 μg/mL vitamin B1, 20 μg/mL tryptophan, and supplemented with the appropriate amounts of antibiotics: carbenicillin (100 μg/ml), chloramphenicol (30 μg/ml), and/or kanamycin (50 μg/ml). Independent clones were first streaked onto solid MOPS-M9 minimal medium. 10-ml cultures in flasks were started at OD_600_ = 0.05 from overnight cultures, and induced eight hours later by addition of IPTG to a final concentration of 0.1 mM. For feeding experiments, 300 μM *p*-coumarate or caffeate was added to the medium at the time of induction. Samples used to analyze tyrosine, anthranilate, *p*-coumarate, caffeate, L-dopa, 3,4,5-trihydroxycinnamateand cinnamoyl anthranilates content were collected after 24 hours of culture. *E*. *coli* BL21(DE3) was used and cultured at 37°C in MOPS-M9 minimal medium containing 1% glucose and carbenicillin (100 μg/ml) or kanamycin (50 μg/ml) for *in*-*vivo* enzyme activities. 10-ml cultures in flasks were started at OD_600_ = 0.05 from overnight cultures of clones containing pSam5 or pRgTAL, and induced and fed 5 hours later by addition of IPTG (0.1 mM) and caffeate (200 μM) or L-dopa (100 μM), respectively. Samples used to analyze 3,4,5-trihydroxycinnamate and caffeate were collected after 24 hours of culture.

**Table 3:** Plasmids and strains used in this study

**Plasmid or Strain**	**Description**	**References**
**Base plasmids**		
pZS21	pSC101; Kan^r^ P_LtetO-1_	[61]
pBbB5a	pBBR1; Amp^r^*lacI* P_lacUV5_	[62]
pBbA5c	p15A; Cm^r^*lacI* P_lacUV5_	[62]
pBbE1a	colE1; Amp^r^*lacI*P_trc_	[62]
pBbE7k	colE1; Kan^r^*lacI* P_T7_	[62]
**Shikimate plasmid**		
pS0	pZS21::*ydiB*-*aroD*-*aro*B-aroG*-*ppsA*-*tktA*	[45]
**Tyrosine plasmid**		
pY	pBbB5a::*tyrB*-tyrA*-*aroC*-*aroA*-*aroL*	This study
**Cinnamoyl anthranilates plasmids**		
pAvn	pBbA5c::*HCBT*-*4CL1*	This study
pAvnD	pBbA5c::*HCBT*-*4CL1*-*tal*	This study
pAvnDF1	pBbA5c::*HCBT*-*4CL1*-*tal*-P_trc_-*sam5*	This study
pAvnDF2	pBbA5c::*HCBT*-*4CL1*-*tal*-P_trc_-*hpaB*-*hpaC*	This study
**Other plasmids**		
pSam5	pBbE1a::*sam5*	This study
pRgTAL	pBbE7k::*tal*	This study
**Strains**		
W3110 *trpD9923*	W3110 [F-λ- INV (rrnD-rrnE) 1] tryptophan auxotroph, randomly mutagenized by treatment with ultraviolet radiation	[41,42]
DH10B	Cloning host	Life technologies
BL21(DE3)	Expression host	Life technologies

### Construction of plasmids

The BglBricks cloning strategy and the BglBricks vectors [62,63] were used for gene assembly. All the forward primers consist of a BglII restriction site at the 5′-end, followed by the Shine-Dalgarno sequence prior to the start codon. The reverse primers consist of the XhoI and BamHI restriction sites at the 5′-end. For the pAvn construct, the gene sequence encoding HCBT (GenBank: CAB06427) from [38] was amplified using the primers HCBTfw and HCBTrv listed in Additional file 2: Table S1. The PCR product was digested with BglII / XhoI and ligated into the pBbA5c plasmid [56] between theBamHI and XhoI restriction sites. The cDNA clone corresponding to *Nt4CL1* (GenBank: U50845) from [48] was amplified using the primers 4CLfw and 4CLrv (Additional file 2: Table S1), digested with BglII / XhoI and ligated into the pBbA5c::*HCBT* construct previously digested with BamHI / XhoI to yield the pAvn plasmid.

For the construction of pAvnD, a gene sequence encoding *Rg*TAL (GenBank: AAA33883) was synthesized (Genescript, NJ, USA) and amplified using the primers TALfw and TALrv listed in Additional file 2: Table S1. The PCR product was digested with BglII / XhoI and ligated downstream *Nt4CL1* into pAvn previously digested with BamHI / XhoI. The *Rg*TAL gene sequence was also ligated downstream the T7 promoter into the pBbE7k plasmid [62] between the BamHI and XhoI sites to obtain the pRgTAL construct.

For the pAvnDF1 construct, a gene sequence encoding Sam5 (GenBank: ABC88666.1) was synthesized (Genescript, NJ, USA) with the BglBricks restriction sites EcoRI and BglII followed by the Shine-Dalgarno sequence at the 5′-end, and with BamHI and XhoI restriction sites at the 3′-end. The *sam5* fragment was released by BglII / XhoI digestions and cloned between the BamHI and XhoI sites of the pBbE1a plasmid [63], downstream the terminator – promoter combination sequence T1-P_trc_, to yield the pSam5 plasmid. The T1-P_trc_-Sam5 fragment was released from pSam5 with BglII / XhoI digestions and ligated downstream *Rg*TAL into pAvnD previously digested with BamHI and XhoI.

For the pAvnDF2 construct, the *hpaBC* operon, which encodes HpaB (GenBank: CAQ34705) and HpaC (GenBank: CAQ34704) was amplified from *E*. *coli* BL21 (DE3) genomic DNA using primers hpaBCfw and hpaBCrv (Additional file 2: Table S1). The PCR product was ligated into the pCR-4Blunt-TOPO vector (Life technologies, Foster City, CA, USA) and a sequenced-verified clone was cured by site-directed mutagenesis to remove an internal BglII restriction site (nucleotides 83–88) using the primers SDM-BglIIfw and SDM-BglIIrv (Additional file 2: Table S1). The cured *hpaBC* operon was cloned into the pBbE1a plasmid downstream the T1-P_trc_ sequence. The T1-P_trc_-*hpaBC* fragment was released with BglII / XhoI digestions and ligated downstream *Rg*TAL into pAvnD previously digested with BamHI and XhoI. For the construction of the pY plasmid, the tyrosine operon in the pY1 plasmid [45] was released with BglII / XhoI digestions and cloned into the pBbB5a plasmid between the BamHI and XhoI restriction sites.

### LC-MS analysis of cinnamoyl anthranilates and precursors

All metabolites were quantified using HPLC–electrospray ionization (ESI)–time-of-flight (TOF) MS. An aliquot of the culture medium was cleared by centrifugation (21,000xg, 5 min, 4°C), mixed with an equal volume of cold methanol–water (1:1, v/v), and filtered using Amicon Ultra centrifugal filters (3,000 Da MW cut off regenerated cellulose membrane; Millipore, Billerica, MA) prior to analysis. For the quantification of intracellular Avn, a cell pellet from 5 ml of culture was washed three times with water, suspended in cold methanol–water (1:1, v/v), sonicated twice for 30 s and centrifuged (21,000xg, 5 min, 4°C). The supernatant was collected and filtered before analysis. The separation of metabolites was conducted on the fermentation-monitoring HPX-87H column with 8% cross-linkage (150-mm length, 7.8-mm inside diameter, and 9-μm particle size; Bio-Rad, Richmond, CA) using an Agilent Technologies 1100 Series HPLC system. A sample injection volume of 10 μl was used throughout. The sample tray and column compartment were set to 4 and 50°C, respectively. Metabolites were eluted isocratically with a mobile-phase composition of 0.1% formic acid in water at a flow rate of 0.5 ml/min. The HPLC system was coupled to an Agilent Technologies 6210 series time-of-flight mass spectrometer (for LC-TOF MS) via a MassHunter workstation (Agilent Technologies, CA). Drying and nebulizing gases were set to 13 liters/min and 30 lb/in^2^, respectively, and a drying-gas temperature of 330°C was used throughout. ESI was conducted in the negative ion mode and a capillary voltage of −3,500 V was utilized. All other MS conditions were described previously [38]. Metabolites were quantified via seven-point calibration curves of authentic standard compounds for which the *R*^2^ coefficients were ≥0.99.

## Abbreviations

Avn: Avenanthramide; DHAvnD: Dihydroavenanthramide D; 4CL: 4-Coumarate/CoA ligase; HCBT: Hydroxycinnamoyl/benzoyl-CoA/anthranilate N-hydroxycinnamoyl/benzoyltransferase; TAL: Tyrosine ammonia lyase; CoA: Coenzyme A.

## Competing interests

JDK has financial conflicts of interest in Amyris, LS9, and Lygos. DL has financial conflicts of interest in Afingen.

## Authors’ contributions

All authors wrote the manuscript. AE and DJ performed the experiments. EEKB conducted the LC-MS analyses. FWC synthesized the Avn D and Avn F standards. DL and JDK supervised the research. All authors read and approved the final version of the manuscript.

## Supplementary Material

Additional file 1: Figure S1 LC-TOF MS analysis of 3,4,5-trihydroxycinnamate produced by engineered *E*. *coli*. **(A)** A sample from the medium of the strain harboring pAvnDF1 after 24 hours of culture. **(B)** Standard, 25 μM 3,4,5-trihydroxycinnamate solution. Click here for file

Additional file 2: Table S1 Primers used in this study. Click here for file
