# Correction: Production of hydroxycinnamoyl anthranilates from glucose in *Escherichia coli*

**DOI:** 10.1186/1475-2859-13-8

**Published:** 2014-01-15

**Authors:** Aymerick Eudes, Darmawi Juminaga, Edward E K Baidoo, F William Collins, Jay D Keasling, Dominique Loqué

**Affiliations:** 1 Joint BioEnergy Institute, Emeryville, CA 94608, USA; 2 Physical Biosciences Division, Lawrence Berkeley National Laboratory, Berkeley, CA 94720, USA; 3 California Institute for Quantitative Biosciences and the Synthetic Biology Institute at UC Berkeley, Berkeley, CA 94720, USA; 4 Eastern Cereal and Oilseed Research Centre, Agriculture and Agri-Food, Ottawa, ON K1A 0C5, Canada; 5 Department of Bioengineering, Department of Chemical & Biomolecular Engineering, University of California, Berkeley, CA 94720, USA

## 

Following publication of this work [1] we noticed that an outdated protocol for metabolite separation has been accidentally described in “LC-MS analysis of cinnamoyl anthranilates and precursors” paragraph of the materials and methods section. The correct protocol is shown below.

## LC-MS analysis of cinnamoyl anthranilates and precursors

All metabolites were quantified using HPLC–electrospray ionization (ESI)–time-of-flight (TOF) MS. An aliquot of the culture medium was cleared by centrifugation (21,000 × g, 5 min, 4°C), mixed with an equal volume of cold methanol–water (1:1, v/v), and filtered using Amicon Ultra centrifugal filters (3,000 Da MW cutoff regenerated cellulose membrane; Millipore, Billerica, MA) prior to analysis. For the quantification of intracellular Avn, a cell pellet from 5 ml of culture was washed three times with water, suspended in cold methanol–water (1:1, v/v), sonicated twice for 30 s and centrifuged (21,000 × g, 5 min, 4°C). The supernatant was collected and filtered before analysis. The separation of metabolites was conducted on the Eclipse Plus Phenyl-hexyl column (250-mm length, 4.6-mm inside diameter, and 5-μm particle size; Agilent Technologies, Santa Clara, CA, USA) using an Agilent Technologies 1200 Series HPLC system. A sample injection volume of 5 μL was used throughout. The sample tray and column compartment were set to 4 and 50°C, respectively. The mobile phase was composed of 10 mM ammonium acetate (Sigma-Aldrich, St. Louis, MO, USA) in water (solvent A) and 10 mM ammonium acetate in 90% acetonitrile and 10% water (solvent B). The mobile phases were made up from a stock solution of 100 mM ammonium acetate and 0.7% formic acid (Sigma-Aldrich, St. Louis, MO, USA) in water. A flow rate of 0.5 ml/min was used, unless stated otherwise. Metabolites were separated via gradient elution under the following mobile phase compositions: 30% B (0 min), 80% B (12 min), 30% B (12.1 min), 30% B (12.5 min), 30% B (15.4 min). The flow rate was increased from 0.5 mL/min at 12.1 min to 1 mL/min at 12.5 min, and held at 1 mL/min for the remaining 2.9 min of the HPLC run. The HPLC system was coupled to an Agilent Technologies 6210 series time-of-flight mass spectrometer (for LC-TOF MS) via a MassHunter workstation (Agilent Technologies, CA, USA). Drying and nebulizing gases were set to 11 L/min and 30 lb/in2, respectively, and a drying-gas temperature of 330°C was used throughout. ESI was conducted in the negative ion mode and a capillary voltage of -3,500 V was utilized. All other MS conditions were described previously [2]. Metabolites were quantified via seven-point calibration curves of authentic standard compounds for which the *R*^2^ coefficients were ≥ 0.99.
