# Supplementary figures and images for: Production of hydroxycinnamoyl anthranilates from glucose in Escherichia coli
Source: Microb Cell Fact. 2013 Jun 28;12:62. doi: 10.1186/1475-2859-12-62 (PMC3716870; doi:10.1186/1475-2859-12-62)

## Slide 1
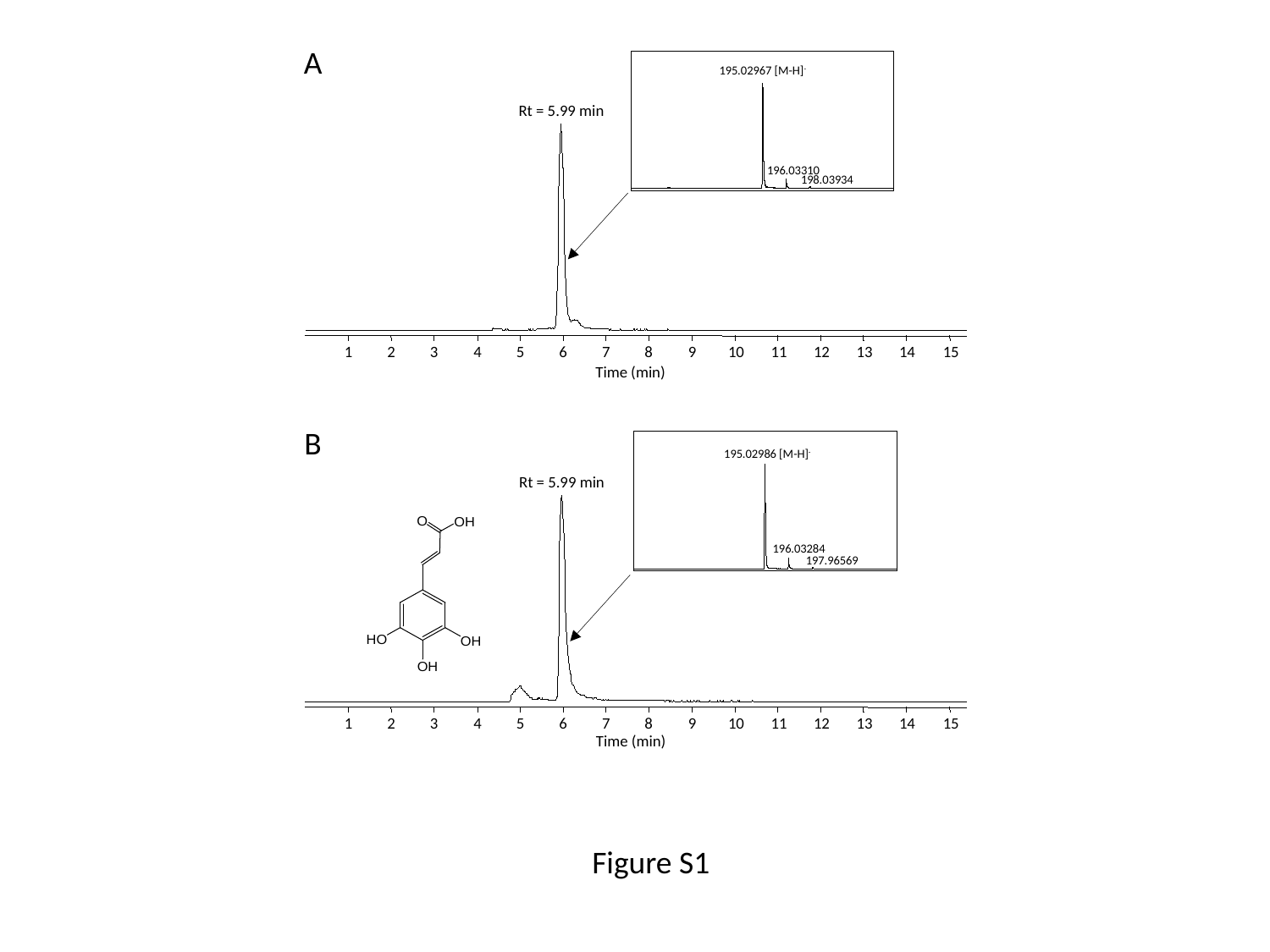

A
195.02967 [M-H]-
Rt = 5.99 min
196.03310
198.03934
1
2
3
4
5
6
7
8
9
10
11
12
13
14
15
Time (min)
195.02986 [M-H]-
Rt = 5.99 min
196.03284
197.96569
1
2
3
4
5
6
7
8
9
10
11
12
13
14
15
Time (min)
B
Figure S1

Supplement: Additional file 1: Figure S1 — LC-TOF MS analysis of 3,4,5-trihydroxycinnamate produced by engineered E. coli. (A) A sample from the medium of the strain harboring pAvnDF1 after 24 hours of culture. (B) Standard, 25 μM 3,4,5-trihydroxycinnamate solution. [file 1475-2859-12-62-S1.pptx]
