# Supplementary material for: Production of hydroxycinnamoyl anthranilates from glucose in Escherichia coli
Source: Microb Cell Fact. 2013 Jun 28;12:62. doi: 10.1186/1475-2859-12-62 (PMC3716870; doi:10.1186/1475-2859-12-62)
Supplement: Additional file 2: Table S1 — Primers used in this study. [file 1475-2859-12-62-S2.docx]

**Table S1.** Primers used in this study

| **Primer name** | **Sequence (5’-3’)** |
| --- | --- |
| HCBTfw | tatatAGATCT*AAAGGAGGCCATCC*ATGTCAATTCAGATCAAGCAG |
| HCBTrv | ATTTACTCGAGAAAGGATCCTTAGAAATCGTAGAAATACTTTTTG |
| 4CL1fw | tatatAGATCT*AAAGGAGGCCATCC*atgccaatggagactactac |
| 4CL1rv | ATTTACTCGAGAAAGGATCCttaatttggaacaccagcag |
| TALfw | tatatAGATCT*AAAGGAGGCCATCC*ATGGCGCCTCGCCCGACTTC |
| TALrv | ATTTACTCGAGGAAAGATCCTTATGCCAGCATCTTCAGCAG |
| hpaBCfw | tatatAGATCT*AAAGGAGGCCATCC*atgaaaccagaagatttccg |
| hpaBCrv | ATTTACTCGAGAAAGGATCCttaaatcgcagcttccatttc |
| SDM-BglIIfw | GCAGGATGGTCGCGAAATCTATATCTATGGCGAGCG |
| SDM-BglIIrv | CGCTCGCCATAGATATAGATTTCGCGACCATCCTGC |

Underlines indicate restriction sites; Italic letters indicate the Shine-Dalgarno sequence.
